# Changes in perfusion and permeability in glioblastoma model induced by the anti-angiogenic agents cediranib and thalidomide

**DOI:** 10.2340/1651-226X.2024.40116

**Published:** 2024-08-14

**Authors:** Jérôme Conq, Nicolas Joudiou, Véronique Préat, Bernard Gallez

**Affiliations:** a Biomedical Magnetic Resonance Research Group, Louvain Drug Research Institute (LDRI), UCLouvain, Brussels, Belgium; b Advanced Drug Delivery and Biomaterials Research Group, Louvain Drug Research Institute (LDRI), UCLouvain, Brussels, Belgium; c Louvain Nuclear and Electron Spin Technologies (NEST) Platform, Drug Research Institute (LDRI), UCLouvain, Brussels, Belgium

**Keywords:** Blood–brain barrier, perfusion, permeability, glioblastoma, anti-angiogenic treatment, DCE-MRI, Evans blue

## Abstract

**Background and purpose:**

The poor delivery of drugs to infiltrating tumor cells contributes to therapeutic failure in glioblastoma. During the early phase of an anti-angiogenic treatment, a remodeling of the tumor vasculature could occur, leading to a more functional vessel network that could enhance drug delivery. However, the restructuration of blood vessels could increase the proportion of normal endothelial cells that could be a barrier for the free diffusion of drugs. The net balance, in favor or not, of a better delivery of compounds during the course of an antiangiogenic treatment remains to be established. This study explored whether cediranib and thalidomide could modulate perfusion and vessel permeability in the brain U87 tumor mouse model.

**Methods:**

The dynamic evolution of the diffusion of agents outside the tumor core using the fluorescent dye Evans Blue in histology and Gd-DOTA using dynamic contrast-enhanced (DCE)-MRI. CD31 labelling of endothelial cells was used to measure the vascular density.

**Results and interpretation:**

Cediranib and thalidomide effectively reduced tumor size over time. The accessibility of Evans Blue outside the tumor core continuously decreased over time. The vascular density was significantly decreased after treatment while the proportion of normal vessels remained unchanged over time. In contrast to histological studies, DCE-MRI did not tackle any significant change in hemodynamic parameters, in the core or margins of the tumor, whatever the parameter used or the pharmacokinetic model used. While cediranib and thalidomide were effective in decreasing the tumor size, they were ineffective in transiently increasing the delivery of agents in the core and the margins of the tumor.

## Introduction

Glioblastoma (GB) is the most aggressive and common malignant primary brain tumor in adults, accounting for more than 50% of all gliomas. Despite an aggressive treatment involving surgical resection (when possible) followed by concurrent radiotherapy/chemotherapy, prognosis for patients with GB remains poor, with a relapse almost inevitable and a median overall survival (OS) of around 10 to 16 months post-diagnosis [1–4]. GB is characterized by a rapid growth, a high degree of cellular and genetic heterogeneity, high infiltrative properties and high angiogenic activity due to excessive levels of pro-angiogenic factors such as members of the vascular endothelial growth factor (VEGF) family [5, 6]. Tumor angiogenesis leads to abnormal tumor vessels that present a tortuous and disorganized structure preventing a proper delivery of the anti-cancer drugs to the tumor site [7, 8]. Another factor that may impair the delivery of chemotherapeutic agents is the blood–brain barrier (BBB). In physiological conditions, the brain vasculature is a highly specialized interface involving three main cell types: endothelial cells, astrocytes, and pericytes. Together, they form and regulate the BBB, which selectively permits the exchange of molecules between the intra-cerebral system and the rest of the body [9]. In GB, even though the BBB is often compromised in the tumor core, the highly permeable leaky vessels contribute to a high interstitial fluid pressure and an impaired delivery of drugs [10–14]. Of note, the BBB generally remains intact or is only slightly compromised in the tumor margins, significantly limiting the passage of drugs to reach the infiltrating tumor cells that will be at the origin of the tumor recurrence [10, 13, 14, 15].

Innovative strategies are emerging to face the problem of impaired drug delivery, including appropriate use of anti-angiogenic treatments that were originally developed to starve tumors. It has been suggested that, during the early phase of an anti-angiogenic treatment, a transient effect of tumor vessels normalization could occur with an improvement in the delivery of anticancer drugs administered during this normalization window [16–20]. The pruning of immature vessels could lead to a remodelling of the tumor vasculature, making it more structured and functional, leading to an improvement in blood perfusion as well as a decrease in tumor hypoxia and intra-tumoral pressure [21–24]. The increase in blood perfusion would increase the supply of anti-cancer drugs at the blood–tumor interface and the decrease in intra-tumoral pressure would facilitate their penetration into the tumor. However, the restructuration of blood vessels could result in an increase in the proportion of normal endothelial cells that could in turn be a barrier for the free diffusion of drugs. The net balance, in favour or not, of a better delivery of compounds in the core and the margins of the tumors, during the course of an antiangiogenic treatment, remains to be established.

This work evaluated whether an anti-angiogenic treatment could alter hemodynamic parameters and the distribution of compounds in the core and the margins of the GB tumor model U87. This model was selected because it presents a well-delineated bulky tumor core, facilitating the analysis of perfusion/permeability in the core and in the margins of the tumor (where infiltrative cells may be present). For this purpose, two different antiangiogenic agents, cediranib and thalidomide, were used because they were previously investigated for a potential normalization effect at an early stage of treatment [21, 25–28]. The analysis included a time-dependent evolution of hemodynamics as the normalization time window could be transient after the initiation of the treatment. Histological analysis was performed with a focus on the capability of the Evans Blue dye to diffuse outside the tumor core and to reach the margins of the tumors. Evans blue, a fluorescent marker of vascular leakage, was used to assess the functionality of the BBB [29]. This dye forms a tight bond with the plasma protein, albumin, in the bloodstream. Albumin, under normal circumstances, is too large to cross the intact BBB . However, in the event of disruption of the BBB, Evans Blue bound to albumin could penetrate the brain parenchyma. The studies were completed by non-invasive DCE-MRI studies to investigate the possibility of this technology to tackle subtle changes in tumor hemodynamics that could be induced by antiangiogenic agents. Studies were completed by immunohistochemistry with the endothelial cell marker CD31 (also known as PECAM-1 or Platelet Endothelial Cell Adhesion Molecule-1), a transmembrane glycoprotein expressed by endothelial cells to assess the evolution of vascular density and vascular integrity in the tumor [30–32].

## Materials and methods

### Orthotopic U-87MG mouse model

All experiments were performed in accordance with the European Directive 2010/63/EU and following the Belgian national regulation guidelines and were approved by the ethical committee for animal care by the Faculty of Medicine of the UCLouvain (2019/UCL/MD/004). Mice were housed under standardized conditions of light and temperature (12-h daylight cycle, 22 ± 2°C) before and during the experiments. They had ad libitum access to chow pellets and water. Animal body weight was constantly monitored throughout the experiment.

Six-week-old female NMRI nude mice (Janvier, France) were anesthetized via intraperitoneal injection of ketamine/xylazine (100 and 13 mg/kg, respectively) and fixed on a stereotaxic frame. In 2 µL of Eagle’s Minimum Essential Medium (EMEM) (Gibco™, ThermoFisher Scientific, Waltham, MA, USA), 4 × 10^4^ cells of U-87MG (ATCC) were injected into the right frontal lobe using an infusion syringe pump (Harvard Apparatus, Holliston, MA, USA) mounted with a Hamilton syringe (26S gauge needle). The injection coordinates were 2.1 mm lateral and 0.5 mm posterior from the bregma, and 2.6 mm deep from the outer border of the cranium [33, 34]. The tumor size monitoring was performed via MRI (see Section 2.3).

### Anti-angiogenic treatment protocol

When the tumor size reached 7 ± 1 mm^3^, treatment was started with either cediranib (Hölzel Biotech, Köln, Germany), racemic thalidomide (Sigma-Aldrich, Saint Louis, MO, USA) or saline (control). Cediranib was administered via oral gavage at the dose of 6 mg/kg each day [35]. Thalidomide was administered intraperitoneally at a dose of 200 mg/kg each day [16, 21]. No signs of adverse events or toxicity were observed during the experiment.

### MRI

MRI was performed using an 11.7 T Bruker Biospec MRI system (Bruker, Ettlingen, Germany) equipped with a ^1^H quadrature transmit/receive birdcage coil (21 mm inner diameter, RAPID Biomedical, Rimpar, Germany). Mice were anesthetized with isoflurane mixed with air (2.5% for induction, 1.5% for maintenance). Animals were covered with a heating blanket and their temperature was monitored. A pressure pad was used to monitor the respiration rate.

Anatomical images were obtained using T_2_-weighted rapid acquisition with a refocused echo (RARE) sequence (echo time = 30 ms; repetition time = 2,500 ms; number of slices = 25; field of view = 20 mm × 20 mm; matrix size = 200 × 200; resolution = 0.1 mm × 0.1 mm; slice thickness = 0.3 mm; acquisition time = 5 min 20 s; averages = 8). Tumor volume was determined from a manually drawn region of interest (ROI) using Paravision 6.0.1 software (Bruker BioSpin) on day 14 following tumor induction, and then daily until the tumor size reached 7 ± 1 mm^3^. After that, anatomical image acquisitions were performed on day 0, 2, 4 and 6 after the beginning of the treatment.

For DCE-MRI acquisition, T_1_-weighted gradient echo images were obtained via a fast low-angle shot (FLASH) sequence (echo time = 1.4 ms; repetition time = 11.719 ms; flip angle = 10.0°; field of view = 20 mm × 20 mm; matrix size = 128 × 128; resolution = 0.156 mm × 0.156 mm; slice thickness = 0.9 mm; averages = 1; total acquisition time = 22 min 40 s). A set of 450 scans with a temporal resolution of 3.02 s was acquired, with Gd-DOTA (Dotarem^®^ 0.5 mol/mL; Guerbet, Villepinte, France) administered intravenously at a dose of 0.29 mmol/kg after the 10th scan over 5 s. DCE-MRI acquisitions were performed on day 0, 2, 4 and 6 after the beginning of the treatment.

### Histology and immunohistochemistry

Evans blue dye (EB: 2% in normal saline; Alfa Aesar, Haverhill, MA, USA) was intravenously injected (3 mL/kg) on day 0, 2, 4 or 6 after the beginning of the treatment. Thirty minutes later, the mice were euthanized and intracardially perfused with paraformaldehyde (PFA) 4% to discard all the remaining dye in the blood vessels and fix the tissue. Brains were then removed, fixed overnight in PFA 4%, cryoprotected in sucrose 20%, included in optimal cutting temperature compound (OCT, Sakura Finetek, Alphen aan den Rijn, The Netherlands), and kept at −80°C. Cryostat 30 µm sections were counterstained with diamidino-2-phenylindole (DAPI, Thermofisher, Waltham, MA, USA) and immunohistologically stained with an antibody against CD31.

For the immunostaining, slides were subjected to antigen retrieval by heating at 98 °C for 30 min in 10 mM citrate buffer (pH 5.7). Endogenous peroxidases were inhibited using 1% H_2_O_2_ and non-specific antigenic sites were neutralized with 5%BSA/TBS/Tween solution. Between all the consecutive steps of the staining procedure, sections were rinsed three times for 3 min in TBS/Tween solution. Primary rabbit anti-mouse CD31 antibody (Cell Signaling Technology, Danvers, MA, USA) diluted 1/150 in 1%BSA/TBS/Tween solution was applied to sections overnight at 4°C. Next, the sections were incubated at room temperature for 40 min with a secondary anti-rabbit antibody (Agilent, Belgium). The slides were then incubated for tyramide signal amplification with Alexa Fluor 488-tyramide reagent (Invitrogen^TM^, ThermoFisher Scientific, UK) diluted 1/200 in 0.1M borate buffer (pH 7.8) supplemented with 0.003% H_2_O_2_, for 10 min at room temperature. Finally, coverslips were mounted with a DAKO fluorescence mounting medium (Agilent, Belgium).

The brain sections were then scanned under a fluorescence microscope slide scanner (Panoramic 250 Flash III, 3DHistech, Budapest, Hungary) with DAPI, Fluorescein Isothiocyanate (FITC) and Cyanine 5 (Cy5) filters [36, 37].

### Image processing

DCE-MRI data were analyzed using in-house software written in Matlab (version 9.6). ROIs were manually delineated. We considered ROI T_1_ as the delineation of the entire tumor area using T_1_-weighted images with Gd-DOTA as the contrast agent, ROI T_2_ as the delineation of the tumor bulk area using T_2_-weighted anatomical images, and ROI Delta as the ROI T_1_ from which we subtracted ROI T_2_ in order to cover the margins of the tumor. In this way, we were able to study the hemodynamic parameters not only for the whole tumor region but also for the margins of the tumor as previously described [34].

The hemodynamic parameters were computed using two different pharmacokinetics models. The first one is the extended Tofts model [38], which is a two-compartmental model that describes a highly perfused tissue, as found in glioblastoma tumors, considering bidirectional transport between the blood plasma and the extracellular extravascular space (EES). The equation of this model is given by:


$$C_{t}\left(t\right)=v_{p}\cdot C_{p}\left(t\right)+K^{trans}\int_{0}^{t}C_{p}\left(\tau \right)e^{-k_{ep}\left(t-\tau \right)}d\tau$$ (1)

where K^trans^ is the volume transfer constant between blood plasma and EES [min^−1^], v_p_ is the blood plasma volume per unit volume of tissue, and k_ep_ is the flux rate constant between EES and blood plasma [min^−1^] [39]. V_e_ is the EES volume per unit volume of tissue, calculated as follows:


$$v_{e}=K^{trans}/k_{ep}$$ (2)

The second pharmacokinetic model used is the Patlak model [40]. This model is comparable to the extended Tofts model but it ignores the backflux from the EES into the blood plasma compartment. Consequently, it only allows for the estimation of the two parameters K^trans^ and v_p_. The equation of this model is given by:


$$C_{t}\left(t\right)=v_{p}\cdot C_{p}\left(t\right)+K^{trans}\int_{0}^{t}C_{p}\left(\tau \right)d\tau$$ (3)

We were also interested in AUC60 and AUC90 corresponding to the area under the curve (AUC) of contrast agent concentration as a function of time from 0 to 60 or to 90 s.

Histological images were analyzed using Qupath (version 0.3.2) [41]. The tumor ROI and the Evans blue diffusion ROI were manually delineated. Tumor vessel density quantification and the percentage of vessels with intact endothelial linings were counted manually.

### Statistical analyses

Data are represented as means ± standard deviation (SD). All experiments were performed in triplicate or more. To test the assumption of normal distribution of the samples, the Shapiro–Wilk test was used. As the test always showed a normal distribution of the data, parametric tests were used. Two-way ANOVA tests (Tukey’s test) and t-tests were performed using GraphPad Prism (version 9.1.2), with *p*-values < 0.05 (*), *p* < 0.01 (**), *p* < 0.001 (***), and *p* < 0.0001 (****) considered as the levels of significance.

## Results

### Histological studies

**Figure 1:**
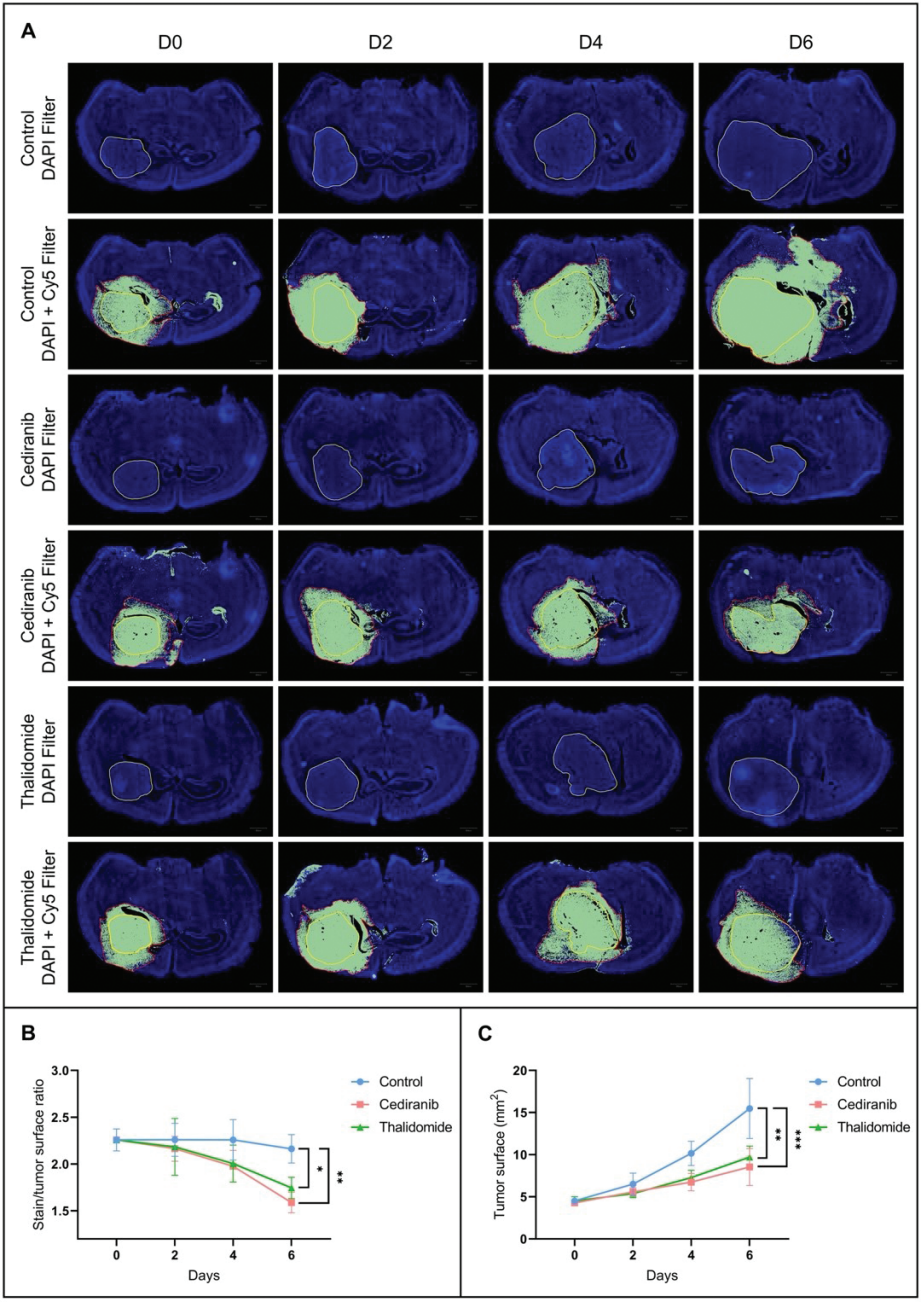
Histological analyses. (A) Representative histological images of brain sections from control mice (*n =* 4) and mice treated with cediranib (6 mg/kg/day, p.o., *n =* 4) and thalidomide (200 mg/kg/day i.p., *n =* 4) on day 0, 2, 4 and 6 after the beginning of the treatment. The use of DAPI filter allows the delineation of tumor area (yellow delineation). The use of Cy5 filter allows the delineation of areas accessible to the Evans Blue dye (delineation in red). (B) Evolution over time of the diffusion of the Evans Blue outside the tumor core. Compared to the untreated group, the fluorescent dye diffused less widely outside the tumor area in mice treated with cediranib and thalidomide. (C) Evolution over time of the tumor size. The tumor size was significantly decreased in both groups treated with anti-angiogenic agents compared to the untreated group. The results are expressed as means ± SD. * *p-*values *<* 0.05, ** *p* < 0.01, *** *p* < 0.001, two-way ANOVA tests (Tukey’s test).

Perfusion and permeability were assessed by perfusing mice with EB dye, a fluorescent vascular leakage marker. The area accessible to the EB dye is larger than the tumor bulk area as measured using DAPI (Figure 1A). On the brain sections of mice treated with anti-angiogenic agents, the fluorescent dye diffused less largely outside of the tumor area in mice treated with antiangiogenic agents compared to the untreated mice (Figure 1A and 1B). On day 6 after the start of the treatment, there was a significant decrease in the EB stained/tumor surface ratio for mice treated with cediranib (1.59 ± 0.11, mean ± SD, *n* = 4, *p* < 0.01) and thalidomide (1.74 ± 0.11, mean ± SD, *n* = 4, *p* < 0.05), compared to the control group (2.16 ± 0.15, mean ± SD, *n* = 4). This result suggests either a decrease in vessel permeability or a destruction of tumor vessels and a consequent decrease in tumor perfusion.

The evolution of tumor size between treated and untreated mice was also studied on brain sections. The tumor size was significantly decreased after 6 days of treatment in mice treated with cediranib (8.51 ± 2.19 mm^2^, mean ± SD, *n = 4*, *p* < 0.001) and thalidomide (9.71 ± 1.29 mm^2^, mean ± SD, *n =* 4, *p* < 0.01) compared to untreated mice (15.48 ± 3.55 mm^2^, mean ± SD, *n =* 4) (Figure 1C).

**Figure 2:**
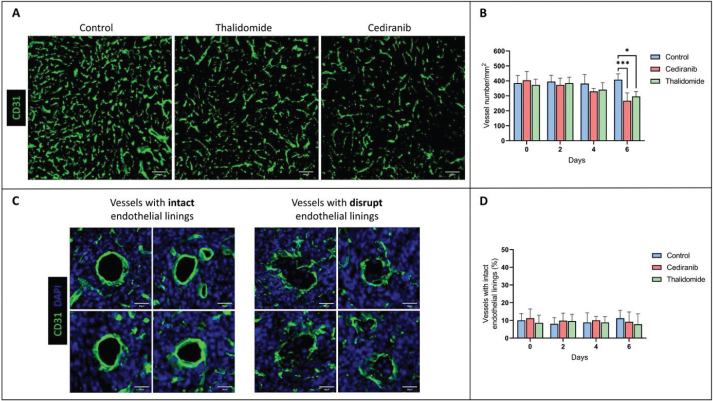
Assessment of tumor vascularity using immune-histological staining for the endothelial cell marker CD31. (A) Representative histological images of brain sections with CD31 labelling from control mice (*n =* 4) and mice treated with cediranib (*n =* 4) and thalidomide (*n =* 4) on day 6 after starting the treatment. (B) Evolution of vascular density over time in control, cediranib and thalidomide-treated mice. Mean ± SD, *n* = 4, *** *p* < 0.001, two-way ANOVA tests (Tukey’s test). The vascular density significantly decreased on day 6 for mice treated with anti-angiogenic agents compared to the control group; (C) Illustration of vessels with intact and disrupted endothelial linings in brain sections with CD31 labelling. (D) Percentage of tumor vessels with intact endothelial linings over time for the control group and groups treated with cediranib and thalidomide. There was no significant difference in the vascular integrity between treated and untreated groups, whatever the day studied. The results are expressed as means ± SD. * *p-*values *<* 0.05, *** *p* < 0.001.

In addition, tumor angiogenesis was assessed by immunochemistry with CD31 labelling, providing parameters for vascular density and vascular integrity (Figure 2A–D). The vascular density significantly decreased on day 6 for mice treated with cediranib (266 ± 53 vessels/mm^2^, mean ± SD, *n =* 4, *p* < 0.001) and thalidomide (296 ± 33 vessels/mm^2^, mean ± SD, *n =* 4, *p* < 0.01) compared to untreated mice (408 ± 39 vessels/mm^2^, mean ± SD, *n =* 4) (Figure 2B). The percentage of vessels with continuous endothelial linings (marker of vascular integrity) is shown in Figure 2C. No significant difference (*p >* 0.05) was observed in the proportion of vessels presenting vascular integrity between the control group (*n =* 4) and groups receiving cediranib (*n =* 4) or thalidomide (*n =* 4), whatever the day studied (Figure 2D).

### MRI studies

#### Diffusion of Gd-DOTA outside the tumor core

**Figure 3:**
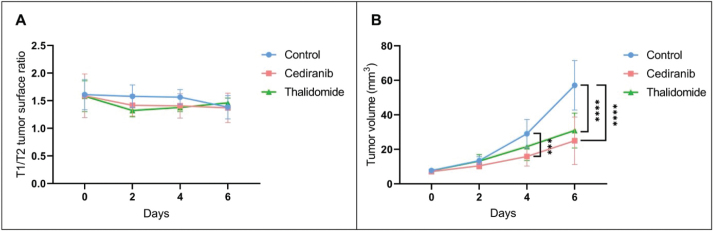
Evolution over time of the diffusion of the contrast agent Gd-DOTA outside the tumor core and of the tumor volume evolution using MRI. (A) Ratio between the T_1_ and T_2_ surface areas reflecting the diffusion of the contrast agent outside the tumor core over time. Measurements were performed on day 0, 2, 4 and 6 after the beginning of the treatment for control mice (blue, *n* = 8) and mice treated with cediranib (red, *n* = 7) and thalidomide (green, *n* = 7). No significant difference was observed between the different groups. (B) Evolution of tumor size over time. Compared to the control group, the tumor size was significantly lower from day 4 for mice treated with cediranib and from day 6 for mice treated with thalidomide compared to the untreated mice. The results are expressed as means ± SD. *** *p-*values *<* 0.001, **** *p* < 0.0001, two-way ANOVA tests (Tukey’s test).

The diffusion of the contrast agent Gd-DOTA outside of the tumor was evaluated by comparing the T_2_-weighted images, corresponding to the anatomical delineation of the tumor core, and T_1_-weighted images corresponding to the area in which the contrast agent was able to diffuse (Figure 3A) [34]. For all tumors, a leakage of the contrast agent outside the tumor core was observed, indicating a high permeability of the vessels and/or a non-integrity of the BBB. There was no significant difference (*p* > 0.05) between the T_1_/T_2_ surface ratio of mice treated with cediranib or with thalidomide compared to the untreated group, whatever the day studied. The analysis of T_2_-weighted images also revealed that the tumor volume evolved differently in the different groups, with a significantly smaller tumor volume from day 4 for mice treated with cediranib (15.81 ± 5.51 mm^3^, mean ± SD, *n =* 7, *p* < 0.001) compared to control mice (29.01 ± 8.29 mm^3^, mean ± SD, *n =* 8) and from day 6 for mice treated with thalidomide (30.88 ± 10.08 mm^3^, mean ± SD, *n =* 7, *p* < 0.0001) compared to control mice (57.09 ± 14.32 mm^3^, mean ± SD) with an even more significant difference on day 6 for mice treated with cediranib (24.94 ± 13.78 mm^3^, mean ± SD, *p* < 0.0001). The decrease in tumor size observed by MRI was consistent with the changes observed by histology.

#### Hemodynamic parameters assessed by DCE-MRI

**Figure 4:**
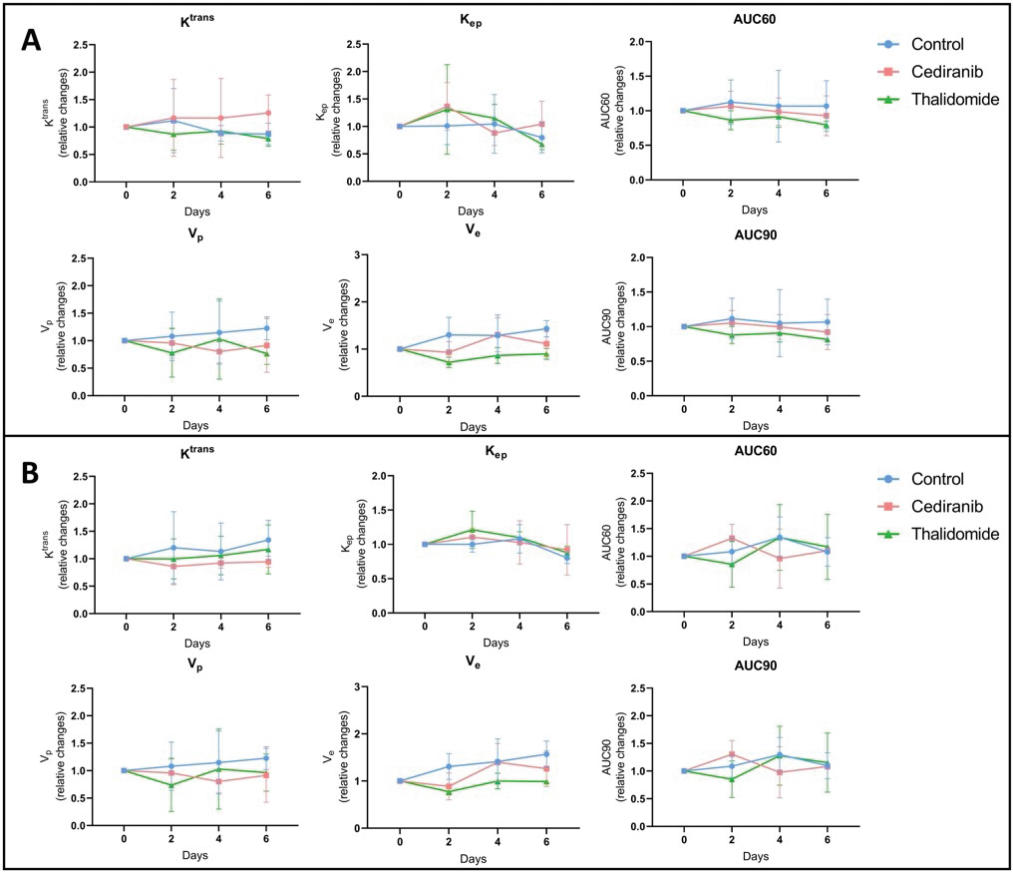
Hemodynamic parameters measured by DCE-MRI computed with extended Tofts pharmacokinetic model. Relative changes in tumor hemodynamic parameters between control group (blue), mice treated with cediranib (red), and mice treated with thalidomide (green). (A) Parameters computed using ROI T_1_, corresponding to the whole tumor area (B) Parameters computed using ROI Delta, corresponding to the margin tumor area DCE-MRI acquisitions were performed just before the beginning of the treatment (day 0), and on day 2, 4 and 6 after starting the treatment. There was no significant difference in the tumor hemodynamic parameters between control group (*n =* 8) and groups receiving cediranib (*n =* 7) or thalidomide (*n =* 7), whatever the studied parameter and ROI used. The results are expressed as means ± SD.

**Figure 5:**
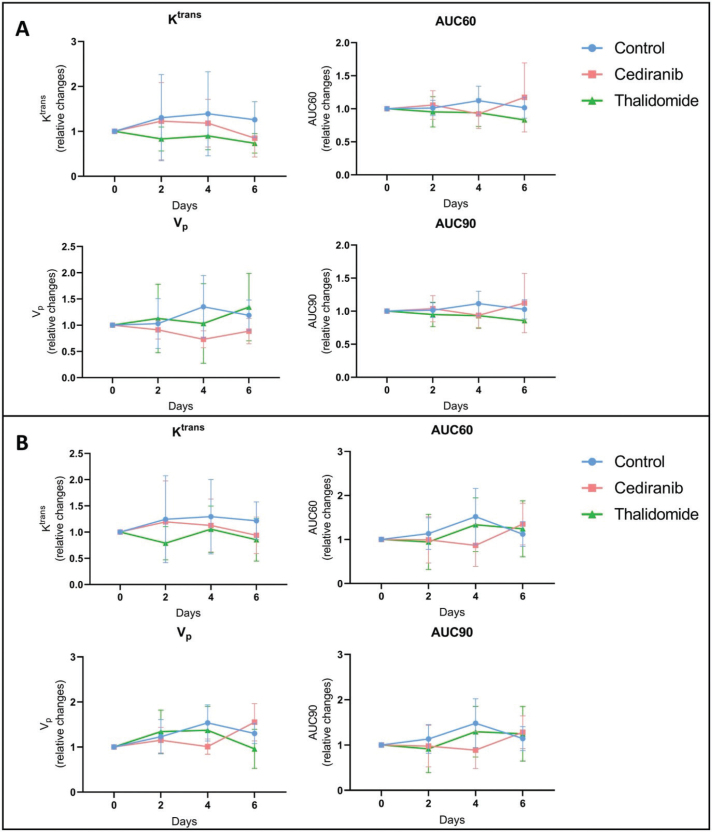
Hemodynamic parameters measured by DCE-MRI computed with Patlak pharmacokinetic model. Relative changes in tumor hemodynamic parameters between control group (blue), mice treated with cediranib (red), and mice treated with thalidomide (green). (A) Parameters computed using ROI T_1_, corresponding to the whole tumor area (B) Parameters computed using ROI Delta. corresponding to the margin tumor area DCE-MRI acquisitions were performed just before the beginning of the treatment (day 0), and on day 2, 4 and 6 after starting the treatment. There was no significant difference in the tumor hemodynamic parameters between control group (*n =* 8) and groups receiving cediranib (*n =* 7) or thalidomide (*n =* 7), whatever the studied parameter and ROI used. The results are expressed as means ± SD.

To further investigate perfusion/permeability, DCE-MRI was performed to provide hemodynamic parameters including the contrast agent efflux transfer constant (K^trans^), the contrast agent reflux transfer constant (K_ep_), the intravascular volume fraction (v_p_), the extravascular volume fraction (v_e_), and the area under the curve of contrast agent concentration as a function of time from 0 to 60 or 90 s (AUC60 and AUC90, respectively) (Figure 4). These parameters were computed using two different pharmacokinetic models: the extended Tofts model [38] and the Patlak model [40]. The extended Tofts model is a two-compartmental model widely used in the literature for studying tumor perfusion/permeability in glioblastoma. The Patlak model is comparable to the extended Tofts model but ignores the backflux and is therefore adapted to assess changes for a low-level BBB permeability where backflux is negligible, as found especially in the margins of glioblastoma tumors. The hemodynamic parameters were analyzed in two different ROIs of the tumor: the ROI T_1_ corresponding to the whole tumor region, and the ROI Delta corresponding to the margin tumor area. No significant difference was observed in the tumor hemodynamic parameters between the control group (*n =* 8) and groups receiving cediranib (*n =* 7) or thalidomide (*n =* 7), whatever the studied parameter (K^trans^, k_ep_, v_p_, v_e_), pharmacokinetic model and ROI (whole tumor or margins) used (Figure 5).

**Figure 6:**
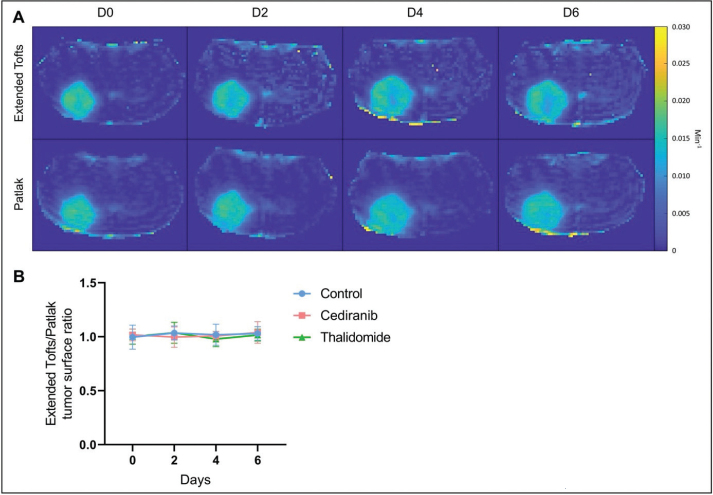
Comparison between K^trans^ parametric maps generated by the extended Tofts model and the Patlak model. (A) Illustrative K^trans^ maps obtained from cediranib treated mouse computed using extended Tofts model (top row) or Patlak model (bottom row). (B) Ratio between the area accessible to the contrast agent using the K^trans^ map from the extended Tofts model and the one measured using the Patlak model. No significant difference was observed over time between these areas. The results are expressed as means ± SD.

K^trans^ parametric maps generated by the extended Tofts model and the Patlak model were compared (Figure 5). The ratio between the area accessible to the contrast agent was compared using the K^trans^ map from the extended Tofts model and the one measured using the Patlak model. This ratio was very close to 1, whatever the treatment taken the day after the beginning of the treatment. There was no significant difference between tumor surface areas measured on K^trans^ maps obtained with the extended Tofts model and those obtained with the Patlak model (Figure 6).

## Discussion

Treating glioblastoma presents a unique challenge in oncology. In addition to many other factors, the poor delivery of drugs to infiltrating tumor cells contributes to therapeutic failure [13, 14, 42]. In this context, strategies to open the BBB and/or increase the drug diffusion inside and outside the tumor core are actively investigated. The use of anti-angiogenic agents to normalize tumor vessels has been suggested as an innovative strategy to improve the delivery of anti-cancer agents in GB [16–20]. It should be emphasized that antiangiogenic treatments have been initially suggested because of the upregulation and activation of VEGF in GB. In recurrent GB, bevacizumab has shown promise in extending progression-free survival (PFS) but with no effect to prolong OS [43–45]. A meta-analysis compared two cohorts of patients treated with thalidomide or bevacizumab. Both drugs showed comparable results in the PFS and 1-year median OS rates [46, 47]. In phase II trials, cediranib, an oral pan-VEGF receptor tyrosine kinase inhibitor, combined to radiation therapy also demonstrated an improvement in PFS but no benefit in OS [48]. This study was focused on thalidomide and cediranib because both compounds were previously suggested to offer a normalization window at the early phase of the anti-angiogenic treatment [21, 25–28]. Theoretically, pruning the most immature vessels should result in a more efficient perfusion. In turn, as the immature vessels coming from angiogenesis are known to offer large fenestrations, their elimination could decrease the permeability and hamper the diffusion of compounds. To establish the resulting balance, we evaluated the distribution of Evans Blue, a marker that integrates both perfusion and permeability. As Evans Blue avidly binds to albumin, it may diffuse in a tissue only if this tissue is perfused and if the vessels are sufficiently permeable, either because the BBB is compromised or because present in regions rich in immature vessels. Even though both treatments (cediranib and thalidomide) were efficient in decreasing the tumor size, contrary to our expectation, we did not observe any increase, even transient, in the diffusion of this marker outside the tumor core during treatment. On the contrary, the stained surface by this marker continuously and significantly decreased over time for treated animals compared to untreated animals (Figure 2). This decrease in Evans Blue extravasation could be due to either a refenestration of the BBB during normalization (with a reduction in the size of the pores between the endothelial cells during vascular restructuring) or a decrease in vessel density due to the antiangiogenic effect, decreasing the amount of Evans Blue able to diffuse into the brain parenchyma. The discrimination between both aspects was clarified by the results obtained using immunochemistry with CD31. A significant decrease in the number of vessels was observed for mice that were treated with anti-angiogenic agents (Figure 2). On day 6, a significantly lower number of vessels was measured in histological sections coming from treated mice compared to untreated mice (Figure 2B). Importantly, no significant change was observed over time in the proportion of vessels displaying vascular integrity whatever the anti-angiogenic agent used or the day studied, with the percentage of vessels with intact endothelial linings remaining around 10% (Figure 2D). Together, the decrease in diffusion of the dye outside the tumor core and the decrease in tumor vascular density with unchanged vascular integrity, pleads for a pruning of vasculature without any obvious normalizing effect. The absence of normalization window in the early phase of the treatment in this study seems contradictory to the fact that thalidomide and cediranib were previously suggested to offer a normalization window [21, 25–28]. Because the normalization has been reported to be dose- and time-dependent, we used the same dose and scheme of treatment than in other studies [16, 21, 35]. Even though histological and MRI analyses were performed every 2 days, no transient increase in contrast agent (Evans Blue or Gd-DOTA) uptake inside and outside the tumor core was observed. This normalization effect could likely depend on the tumor model used. The U87 GB model was selected because changes in permeability induced by osmotic shock were previously demonstrated in this model [34]. In the future, it could be interesting to analyze these effects in other glioblastoma models. It could be also interesting to analyze hemodynamics changes every day instead of every other day so as not to miss a possible increase in the delivery of agents. Finally, it could be also interesting to compare the results from this study with cohorts treated with bevacizumab, the antiangiogenic agent presently used in the clinic to treat patients with recurrent GB.

Lessons from the MRI study also deserve commentary. Contrary to histological studies, MRI is fully non-invasive and could potentially be used as a marker of early changes in hemodynamic parameters that could be translated in patients for guiding treatment. A significant decrease in tumor size over time was observed for mice treated with cediranib and thalidomide, indicating the efficacy of those treatments. The MRI results are fully consistent with those obtained by histology. However, none of the parameters tested, K^trans^, k_ep_, v_e_, v_p_, AUC60 and AUC90, in the core and margins of the tumor, were significantly modified during the course of treatments using cediranib or thalidomide compared to untreated mice (Figures 4–5). By analyzing many different parameters, we hypothesized that DCE-MRI could provide more information about subtle changes linked to perfusion and permeability. As none of the parameters changed, we cannot conclude about real hemodynamic changes induced by the treatments on the sole basis of MRI. We may wonder why DCE-MRI (with Gd-DOTA) did not provide the same results as histology (with Evans Blue) regarding the ability of the marker to diffuse outside the tumor core. Indeed, histology suggested a decreased ability to diffuse outside the tumor core while no change in diffusion outside the tumor core was found in MRI. The disparate results observed between MRI and Evans Blue staining to assess vessel permeability could likely be attributed to fundamental differences in the molecular and distribution properties of the contrast agents used for each technique. Indeed, Evans blue (960 Da, molecular weight) is a fluorescent dye that binds to albumin, a plasma protein with a high molecular weight (68 kDa), whereas Gd-DOTA is a small hydrophilic complex (MW = 580 Da). As Evans blue is bound to albumin, it can only cross highly permeable vessels (i.e. in areas with compromised BBB or area rich in immature vessels coming from angiogenesis. In turn, Gd-DOTA, because of its small size, can diffuse more easily into the brain parenchyma even through a weakly compromised BBB. Focusing on the tight junctions between endothelial cells, we can assume that the observed change in permeability would be more pronounced for larger molecules (complex Evans Blue – albumin) than for smaller molecules (Gd-DOTA) that were already able to cross small fenestrations of the damaged tumoral BBB. In future studies, it would be interesting to use Gd-based contrast agents with high affinity for albumin for the MRI method, such as gadobenate (Gd-BOPTA), which would better mimic the distribution behaviour of Evans Blue.

This study has several limitations that deserve further discussion. A first limitation is linked to the orthotopic U-87MG glioblastoma model used in this study. This model was selected because its tumor core is well delineated, rendering easier the assessment of the diffusion of tracers in the margins of the tumor. However, being weakly infiltrative may also be considered as a disadvantage because it does not adequately represent the highly infiltrative biological properties of human glioblastoma [49]. Another limitation of this study is that cediranib and thalidomide were not used in combination with different chemotherapies. Temozolomide (TMZ), the chemotherapy used in the standard of care would not have been relevant in this study, given that it already crosses the BBB very easily. It is important to notice that the results of a phase III trial on 751 patients (CATNON trial) showed that TMZ did not add any benefit compared with radiotherapy alone for the treatment of IDH wildtype glioblastoma [50]. Considering that TMZ drug has very limited benefit and induce significant side effects (myelotoxicity, nausea, vomiting, ulcers, fatigue), this should stimulate new research for treating glioblastoma with other anti-cancer drugs. For example, interesting results have been obtained with paclitaxel and doxorubicin in pre-clinical models of glioblastoma [14, 51]. However, the limited ability to cross the BBB impedes reaching a sufficient therapeutic concentration in the tumor. In the future, it would be worth testing if tumor vessel normalization could improve the delivery of such chemotherapeutic agents. In addition, it is worth mentioning that other strategies exist to modulate the passage of drugs into the brain parenchyma. Among the various BBB-opening strategies, we can mention osmotic shock [34], radiotherapy [52] and focused ultrasound [53].

## Data Availability

All data will be provided upon e-mail request.
